# A review on trade-offs at the warm and cold ends of geographical distributions

**DOI:** 10.1098/rstb.2021.0022

**Published:** 2022-04-11

**Authors:** Yvonne Willi, Josh Van Buskirk

**Affiliations:** ^1^ Department of Environmental Sciences, University of Basel, 4056 Basel, Switzerland; ^2^ Department of Evolutionary Biology and Environmental Studies, University of Zürich, 8057 Zürich, Switzerland

**Keywords:** climate gradients, ecological niche, genetic correlation, limits to adaptation, ‌range limit, thermal stress

## Abstract

Species’ range limits are ubiquitous. This suggests that the evolution of the ecological niche is constrained in general and at the edges of distributions in particular. While there may be many ecological and genetic reasons for this phenomenon, here we focus on the potential role of trade-offs. We performed a literature search on evidence for trade-offs associated with geographical or elevational range limits. The majority of trade-offs were reported as relevant at either the cold end of species' distribution (*n* = 19), the warm or dry end (*n* = 19) or both together (*n* = 14). One common type of trade-off involved accelerating growth or development (27%), often at the cost of small size. Another common type involved resistance to or tolerance of climatic extremes that occur at certain periods of the year (64%), often at the cost of small size or reduced growth. Trade-offs overlapped with some of the classic trade-offs reported in life-history evolution or thermal adaptation. The results highlight several general insights about species' niches and ranges, and we outline how future research should better integrate the ecological context and test for the presence of microevolutionary trade-offs.

This article is part of the theme issue ‘Species’ ranges in the face of changing environments (Part II)’.

## 1. Motivation for searching for trade-offs determining species' distribution

Species have spatially restricted distributions. While ecologists work with a robust framework that can explain species' distribution, evolutionary biologists have not developed a unifying theory. Ecologists propose that species occurrence is explained—when not by dispersal limitation—by the ecological niche ([1, pp. 25–53], [2]). The niche is defined by the environmental conditions that allow populations to persist along gradients of abiotic conditions, resources and densities of other species [3]. Therefore, while an important challenge in understanding distribution limits for ecologists is to define the parameter space that encompasses the niche, evolutionary ecologists face a different problem. They need to explain why niche evolution is constrained in general, and at range margins in particular. Both questions are unresolved. The causes of restricted distributions count among the more important knowledge gaps in evolutionary ecology [4,5].

There are many potential sources of constraint to niche evolution at range limits. Eco-evolutionary theory points to thinning of habitat and lower carrying capacity of the habitat towards range edges, and this reduces demographic rates through genetic drift and mutation accumulation [6]. Another set of problems is related to selection and dispersal. Steep and multivariate gradients produce a high demographic toll by selection [7,8]. Dispersal may improve adaptation with the input of recruits and genetic variation into marginal populations [9,10]. However, too much dispersal can swamp the gene pool of marginal populations with maladaptive genetic variation [11]. While different predictions may apply to particular range limits, the ubiquity of range limits across organisms differing in a variety of attributes—habitat at range limits, the importance of genetic drift, steepness of environmental gradients, dispersal and others—suggests that further, rather general factors may be at play. One such factor may be trade-offs. MacArthur [12, pp. 127–131] noted the relevance of trade-offs in the context of range limits in broad terms, suggesting that certain adaptations make a species successful within its range but constrain occurrence outside of the range. More generally, trade-offs that involve optimization balances between traits operating within (range edge) populations may limit species' ranges. Trade-offs have not commonly been studied empirically in the context of range limits but are known to be prevalent in the evolution of life histories (see below). The goal of this study was to provide an overview on reports on trade-offs associated with range limits in the literature and to synthesize emerging patterns and challenges.

Box 1. **Range limits and trade-offs.**

The geographical distribution of species is typically underlain by environmental gradients covering either the entire distribution or a fraction of it (drawing (*a*)). At some point of a gradient, the species may stop occurring (*b*). When range limits reflect niche limits, it means that the performance of the species becomes too low under the prevailing environmental conditions (*c*). Even though there may be expression of higher resistance, tolerance or avoidance of extremes towards range edges, they become too costly (*d*).

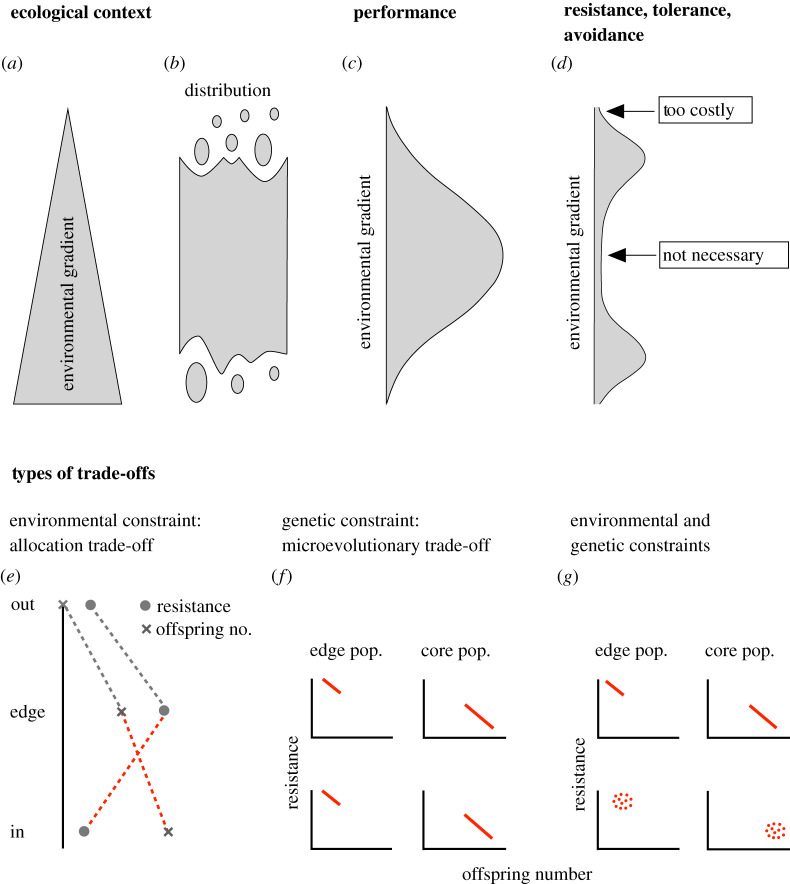

A purely phenotypic, allocation trade-off could contribute to range limits (*e*). Let us assume that it is among resistance to an environmental extreme and offspring number. There may be genetic variation for these traits and that is independent from each other. From a genetic point of view, both traits could therefore simultaneously respond to selection. However, they stand in intra-organismal allocation competition with each other for resources (or energy or time). A response to selection in one trait, e.g. increased resistance at the range edge, implies the increased drawing from the shared pool of resources and leaves less for the other trait, e.g. offspring number, which is also relevant for fitness. The way out would be the evolution towards improved acquisition. The drawing presents a case in which resources are limited at the range edge, and even more so beyond the range edge.

A genetic or microevolutionary trade-off is present between traits when they are genetically non-independent in a way that is antagonistic in regard to fitness (*f*). When selection acts, the traits cannot easily respond to selection in an adaptive direction. Instead, when one trait, e.g. resistance—to stay with the same traits, responds in an adaptive direction, the others are pulled in a maladaptive direction, e.g. towards fewer offspring. Therefore, the adaptive response is slowed, at best. Populations of the edge and core may differ in the position of the trade-off in multivariate trait space (position of the red line in (*f*)), but the shape should be similar among them, and among environments, e.g. in a transplant study with sites within (lower panels) and at the edge of the range (upper panels).

Finally, genetic constraints and environmental constraints may act together (*g*), e.g. such that the genetic trade-off is expressed under limited resources at range edges but not under more favourable conditions in the range centre.

Experimental approaches to estimate the genetic and/or environmental contributions to a trade-off that potentially supports range limits include selection experiments, comparisons among relatives and, most importantly, direct manipulation of resources. Also feasible are long-term studies linking pedigree information with (the co-inheritance of) phenotypic traits in natural populations. Complications in detecting trade-offs (and other important aspects of trade-offs) were outlined by Roff & Fairbairn [20].

## 2. Trade-offs and life-history evolution

In his book on life-history evolution, Stearns [13, pp. 72–90] defines a trade-off as ‘the linkages between traits that constrain the simultaneous evolution of two or more traits'. He lists three types of trade-offs that are non-exclusive: physiological or allocation trade-offs, microevolutionary trade-offs and macroevolutionary trade-offs. Physiological trade-offs appear when energy allocations between two or more functions compete for the same resources within an individual. These may or may not have a proximate genetic basis. Microevolutionary trade-offs not only include physiological trade-offs with a genetic basis but also include all situations in which a change in one trait that increases fitness is genetically correlated with a change in another trait that decreases fitness. Macroevolutionary trade-offs are the negative association between fitness-relevant traits among phylogenetic lineages, sometimes associated with habitat. They are assumed to go back to physiological and microevolutionary trade-offs that were enforced over long periods of time while macroevolutionary patterns among divergent taxa evolved. Comparative analyses allow macroevolutionary trade-offs to be detected even when variation for the trade-off within populations of species has vanished or even changed sign (e.g. [14]).

The genetic cause of correlation is pleiotropy or linkage, although trade-offs caused by the latter are potentially more transient. Linkage is when alleles do not segregate independently because of physical proximity on chromosomes. Pleiotropy is when a gene affects more than one trait. A genetic correlation caused by linkage or pleiotropy is then the net phenotypic effect of all loci that are linked or act pleiotropically. In other words, e.g. a single pleiotropic effect does not necessarily affect the measurable genetic correlation; this is the case only if the locus has major effect. Furthermore, the effect of the environment can be overriding, causing the phenotypic pattern to change from the genetic pattern.

In quantitative genetics, the correlation of the breeding values of traits and the environmental correlation (owing to all other effects) can be readily assessed [15, pp. 312–323]. When these two components of the phenotypic correlation align in sign and magnitude, one suspects identical physiological mechanisms. However, estimating genetic correlations via the resemblance among relatives is associated with large sampling errors. Furthermore, trade-offs detected this way are often unstable across environments [16]. The genetic correlation can also be revealed by selection on one character while assessing the correlated response in another trait, if the heritabilities of both traits are known [15, p. 317].

An extension of the genetic model introduced above is that of antagonistic pleiotropy across habitats. An allele that is beneficial and under positive selection in one habitat may be deleterious in another habitat and may lead to divergent local adaptation and ecotype formation [17]. On the practical side, the methods described above can be applied if the same trait in the two habitats is modelled as two different traits [15]. Such a trade-off may contribute to species' range limits in the case it operates at the limit and beyond, if the edge population has adapted to a local trait optimum and the optimum beyond the edge is very different (e.g. because another, but non-independent strategy is favoured).

Trade-offs among life-history components have been studied for decades. At the time, Stearns [13] reported five trade-offs to be well supported, with the (phenotypic) one between growth and reproduction being the best supported. Evidence in favour of general support for trade-offs came also from species comparisons analysed in the context of alternative life-history strategies such as *r*- versus *K*-selection [18] or *C*-, *S*- and *R*-selection in plants [19], to name two early ones. Trade-offs between growth, reproduction and maintenance to cope with stress may be of particular relevance at species' range limits. Stressful environmental conditions may favour a state of no or low growth and development accompanied with fast growth and development when conditions are favourable (stress avoidance). Alternatively, higher investments into maintenance and protection (stress tolerance or resistance) may be favoured, at the cost of reduced performance in other aspects of fitness. Box 1 illustrates in more detail how trade-offs may contribute to range limits.

## 3. Literature search on trade-offs

We searched the Web of Science for articles reporting trade-offs related to range limits on 10 November 2021. We searched for the term combination of ‘(trade-off* OR tradeoff*) AND (geograph* OR latitud* OR longitud* OR elevation* OR altitud* OR south* OR north* OR east* OR west*) AND (distribution* OR range*) AND (limit* OR edge* OR margin* OR boundar* OR border*)' applied to abstracts or titles of articles, and we excluded articles of the field of Meteorology Atmospheric Sciences. The search revealed 664 entries, of which 119 seemed to contain relevant data based on information in the abstract. Those articles and 29 more (which were referred to in primary articles or found in additional searches) were read carefully. We ended with 41 studies containing information on trade-offs between pairs of traits or trait complexes (*n* = 52) in the context of an environmental gradient associated with range limits. We excluded studies on species with reportedly expanding ranges. Furthermore, studies were excluded when evidence for a (macroevolutionary) trade-off was gathered on the level of species, but only two species were considered; an exception was a study on experimental hybrids of two *Mimulus* species with divergent elevational distribution, selected under low- and high-elevation conditions [21]. Furthermore, studies were also excluded when the gradient reflected a transition between habitat types (e.g. freshwater versus brackish water, tidal zones, etc.) instead of conditions spanning over the geographical range edge. However, we included (and searched for) elevational gradients assuming parallels with geographical (latitudinal) gradients even though low- and high-elevation ends of distribution do not typically reflect outer geographical range edges. Table 1 summarizes the studies.

**Table 1.** Summary of trade-offs reported in the literature in the context of geographical and elevational range limits of species or groups of species. (N, north; S, south; I, insect; P, plant *sensu lato*; Ph, plant, herbaceous; Pt, plant, tree; V, vertebrate; pop, population; CG, common garden; GH, greenhouse; M^+^, manipulation performed*.* Traits written in italics were not measured as such, but the trade-off was hypothesized based on results. Life-history traits are indicated in green, traits related to coping with climatic stress in blue, and traits related to coping with biotic stressors in purple.)

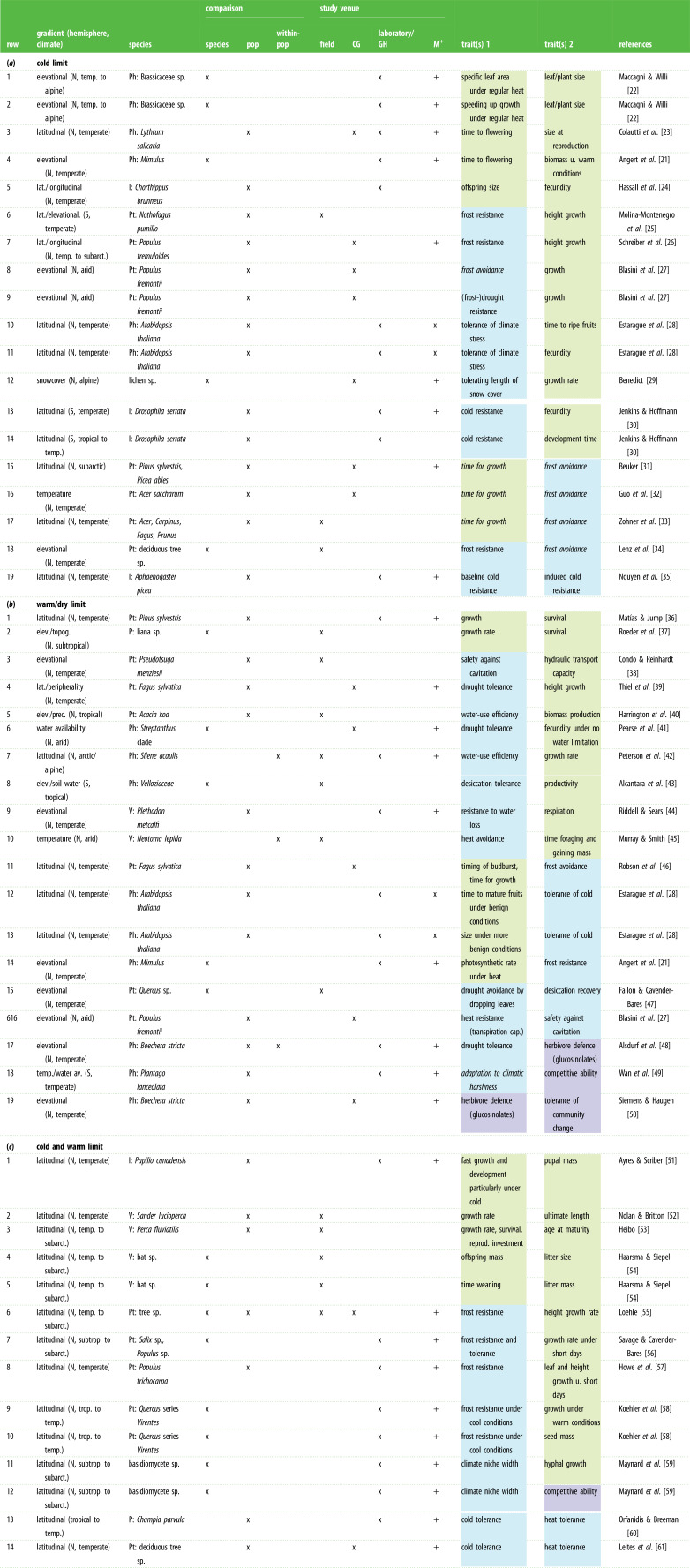

Nearly all studies addressed explicitly latitudinal or elevational gradients and had a focus on temperature or water availability. We therefore categorized trade-offs as being relevant for the cold end of distribution (mostly high latitude, high elevation; *n* = 19), the warm or dry end of distribution (mostly low latitude, low elevation; *n* = 19) or both ends (*n* = 14). In some cases, authors were explicit in regards to which end(s) of distribution trade-offs were more relevant; in others, we judged based on information in the papers. Studies included field surveys with no experimental manipulation (*n* = 13 trade-offs), common garden or laboratory experiments (*n* = 38), or both (*n* = 1). Some of the reported relationships were therefore purely phenotypic, while others were genotypic as the effect of the environment was controlled for to some extent. Most studies were based on the comparison of populations within species (n= 31 trade-offs), others on the comparison of species (*n* = 17) or families within populations (*n* = 2), and two studies considered comparisons on more than one of these levels.

There were obvious biases in the pool of studies considered. More than half of the reported trade-offs were found in plants (one in a red alga, 37 in seed plants), the others in lichens (one), basidiomycetes (two), insects (five), fishes (two), an amphibian (one) and in mammals (three). All except six trade-offs were reported for the Northern Hemisphere. Furthermore, about half of the trade-offs were found in regions of the temperate zone (*n* = 27), fewer in regions with (or including) a subarctic or arctic/alpine climate (*n* = 13) or a (sub-)tropical climate (*n* = 10, of which three extended from subtropical to subarctic areas) and five trade-offs were found in predominantly arid regions.

Studies commonly reported on tolerance or resistance, apart from avoiding stress. We used the term *tolerance* to depict the coping with stress by maintaining growth and reproduction and *resistance* to depict the coping with stress by preventing damage. Often we adopted the terms the authors used, unless their use clearly differed from our definitions.

## 4. Trade-offs at the cold end of distribution

Of the 19 trade-offs found to play a likely role at the cold end of distribution, five were between pairs of life-history (-related) traits, eight between coping with thermal stress and a life-history trait and two between pairs of thermal stress-performance traits (table 1*a*). No study addressed whether trade-offs were microevolutionary, existing within populations with a genetic basis. Therefore, we describe trade-offs by positioning trait combinations of populations within species, or species (for macroevolutionary trade-offs) relative to the environmental gradient, and we summarize (most of) them below in sequence of listing in table 1*a*.

Three studies on plants reported that those living towards the cold end of the gradient grew or developed faster, at the cost of small final size and (presumably) reduced reproductive output (table 1*a*, rows 1–4). Eight trade-offs suggested that coping better with negative temperatures or a long snow cover implied less growth in trees and lichens, and low fecundity and a long development time in *Arabidopsis thaliana* and a *Drosophila* species [26,27,29–34]. An additional three studies on trees reported that populations occurring towards the cool end of the gradient had budburst or leaf-out dates in spring at lower cumulative temperature, probably to lengthen the time for growth and reproduction, at the cost of some damage under likelier frost events [35–37]. However, the trees at the cold end may not experience frost damage because they may have higher frost resistance during that stage, as found in another study on trees [38]. Hence, the foremost environmental trade-off between time for growth/reproduction and frost avoidance seems to be mediated by frost resistance. An evolutionary limit may be achieved by selection for higher frost resistance early in the season while completing growth and development during a short growing season.

In summary, fast growth or development (7 out of 19), or tolerating or resisting the cold (10 out of 19) may often be favoured at the cold end of distribution, which produces costs in other aspects of performance. Trade-offs often involve trait pairs, but sometimes it seems that there is only one, namely the start of vegetative growth, that determines both the length of the season available for growth and reproduction and the chance of being exposed to frosts.

## 5. Trade-offs at the warm end of distribution

Some studies emphasized the warm or dry end of distribution. Of the 19 trade-offs found, two were between pairs of life-history traits, 12 between coping with drought or thermal stress and a life-history trait and two between pairs of traits related to coping with heat or drought (table 1*b*). Three additional trade-offs were reported that included performance under biotic interactions. Three trade-offs were reported within population(s).

Two studies found that plants of the warm or dry end of the gradient grew faster, at the cost of lower survival (table 1*b*, rows 1, 2). Eight trade-offs involved higher tolerance or resistance to dry conditions or heat avoidance, while resource acquisition, production, growth, fecundity, respiration or activity was lower [24–30]. These trade-offs were reported for plants, a salamander and a mammal. Three studies on plants discovered trade-offs between early phenology, fast development or good acquisition capacity under higher temperatures, at the expense of avoiding, tolerating or resisting low temperatures [31–34]. In a comparison of oak species along an elevational gradient, drought avoidance by dropping leaves was more pronounced in low-elevation species, but their leaves had a lower desiccation recovery [35]. A trade-off between traits related to coping with climate stress was also found for a desert *Populus* species, in which low-elevation plants had a higher transpiration capacity to cool leaves, at the expense of a higher risk of cavitation [36]; it is another example where the same trait, here vessel architecture, causes a trade-off.

In the herbaceous plant *Boechera stricta*, two types of trade-offs involving the coping with biotic stressors were relevant at the low-elevation limit. First, there was a trade-off between drought tolerance (root : shoot ratio) and glucosinolate production [37]. Glucosinolates protect the plants against generalist herbivores, which are more problematic beyond the low-elevation range limit [62], but those plants with higher expression are less drought tolerant. The second trade-off was between defence and coping with a change in plant community, and may be relevant even when conditions are not dry. Inbred lines with higher glucosinolate expression were less tolerant of a change in plant species composition across the low-elevation range boundary [39]. Finally, a study on *Plantago lanceolata* reported another trade-off involving a biotic stressor, between adaptation to drier and thermally more variable conditions and competitive ability [38].

In short, fast growth and development (4 out of 19), direct avoiding (2 out of 19), or tolerating or resisting drought or heat (10 out of 19) seem often selected for at the warm and/or dry end of distribution, at the expense of other traits affecting performance. Furthermore, some trade-offs involved the coping with biotic stressors (3 out of 19).

## 6. Trade-offs at the warm and cold end of distribution

A number of studies detected trade-offs across the entire distribution from the cold to the warm end and emphasized that trade-offs occurred between coping between these extremes. Of the 14 trade-offs reported in total, five were between pairs of life-history traits, six between thermal or climatic performance and a life-history trait, two between pairs of performance traits under cold compared to warm conditions and one between climatic tolerance and competitive ability (table 1*c*).

The trade-offs involving life-history traits only were found in animals, in a butterfly, two fish species and in bats (table 1*c*, rows 1–5). They included classic life-history trade-offs involving the rate of growth or aspects of reproduction (one trade-off occurred between both ends and core [52]). In a now classic study, Loehle [55] documented that frost resistance in trees trades off against height growth [26]. He hypothesized that high-latitude limits may be set by frost resistance and low-latitude limits by competitive ability determined by height growth. However, the data did not provide any direct evidence that reduced height growth resulted in reduced competitive ability at the warm end of distribution, nor have three similar studies [27–30]. However, a laboratory study on saprotrophic basidiomycetes described a trade-off between climate niche width and both hyphal growth and competitive ability [31,32]. Finally, in a study on a red alga and another on trees, trade-offs were detected between tolerance of cold and tolerance of warm/hot conditions [33,34].

In conclusion, trade-offs interpreted as relevant across the entire distribution were similar to those reported as relevant for the cold end or warm end of distributions. Conditions at the cold compared to the warm end of distribution seem to select for fast growth (1 out of 14) or thermal (or climatic) tolerance or resistance (9 out of 14), at the expense of other traits affecting performance.

## 7. Synthesis of reported trade-offs

**Figure 1.**
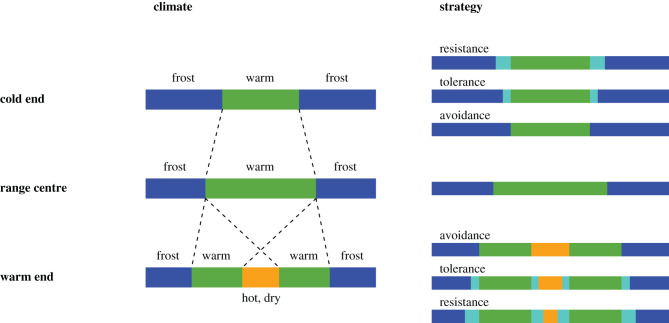
Yearly climate progression across a species' range in the temperate and boreal zones (left) can be often split into two to three main parts: a long winter shouldered by periods of frost events in spring and autumn (ends of bar/blue) and a short growing season with benign conditions (central part/green) at the cold end; a shorter winter with periods of frost and a longer growing season in the range centre; a brief winter and a growing season that is interrupted by a period of hot, dry or hot-dry conditions (very centre/yellow) at the warm end. The dotted lines indicate the relative position of the beginning and end of the benign/growing season. Many other seasonal regions of the world experience a similar pattern of conditions. Accordingly, strategies (right) to cope with the short benign seasons for growth, development and reproduction at the cold and warm end of distribution may involve: avoidance, which requires rapid growth and development, or tolerance of frost and heat and consequently some lengthening of the time for growth and development (inserts/cyan), or resistance to frost and heat and considerable lengthening of the time for growth and development. Mixed strategies are not shown here. (Online version is in colour.)

Two aspects of the trade-offs reported so far are striking at first glance. First, the great majority are related to coping with abiotic stress, either directly or indirectly by their association with the time of the year that has less abiotic stress and better conditions for growth, development and reproduction. In this context, it is noteworthy that most studies come from the temperate or boreal zone or from mountains covering similar climates over a shorter spatial scale. In these regions, yearly climatic progression can be split into four periods: winter, spring during which frosts are likely, a period with more benign temperatures in summer, and an autumn during which the risk of frost returns (figure 1, left). At the cold end of distribution, winters shouldered by frosts in spring and autumn are longer, and the period with more optimal thermal conditions for growth and reproduction is short. At the warm or dry end of distribution, winter is short or absent but the warm summer may have a period that is too hot, hot–dry or dry for growth, development and reproduction. Most trade-offs can be positioned in terms of how organisms cope with changing conditions and durations of these periods of the year.

Different types of strategies may be favoured under the different settings. At the cold end of distribution with a long period of snow and frosts and a short period of warmth, species may cope in one of three ways: frost resistance, frost tolerance or frost avoidance, with the increasing importance of fast growth and development (figure 1, right). Rapid growth and development makes it possible for organisms to finish vital processes during the short warm period, while periods of frosts are avoided. Frost tolerance also must be coupled with considerable speed of growth and development because otherwise the organism cannot compensate for damage caused by frost. Only frost resistance may appreciably lengthen the time available for growth and development. At the warm end of distribution, hot–dry summers may also require either avoidance, tolerance, or resistance to heat and drought. In principle, the same constraint as for the cold end affects the remaining short periods of more optimal growing conditions, with heat or drought avoidance causing the strongest constraints on time available for growth, development and reproduction.

The second striking aspect of the trade-offs in table 1 is that most involve life-history strategies that make sense in the context of the climate peculiarities of cold and warm ends of species' distribution. Rapid growth or development (including phenology) seem often favoured at range edges (27%) and come with intrinsic costs—small size [21–23,51], reduced fecundity [23], short life expectancy [36,37] or reduced tolerance of cold [28]. Resistance in particular can lengthen the time for growth and reproduction considerably [34]. Trade-offs that involve tolerance or resistance to climatic extremes (64%) cause performance declines in other life-history traits: slow growth or reduced size [25–27,29,39,40,42,55–59], slower physiological activity [38,43,44], slower development [28,30], reduced fecundity [28,30,41], reduced ability to handle other or opposite climate stress [27,60,61], reduced defence [48] or reduced competitive ability [49,59]. The majority of trade-offs can be summarized as either typical life-history trade-offs or trade-offs related to thermal/climate adaptation.

## 8. Where to next? Understanding the climate niche and its limits

The studies available so far are admittedly heterogeneous, but they point to insights that agree well with recent ecological and evolutionary studies on species' distributions and climate adaptation. We begin with the general statement that climate is important at many range limits. A recent meta-study on transplant experiments including beyond-range sites in animals and plants revealed that 98% of range limits were associated with a decline in climate suitability [63]. Indirect evidence for climate limitation comes from the many studies reporting range shifts associated with climate change, frequently up in elevation or latitude [64]. A second general statement is that the evolution of the climate niche is limited. Linked with recent climate warming, retractions at the warm edges of species' distributions are as great in magnitude as expansions at the cool edges (e.g. [65]), indicating insufficient adaptation at warm edges [66]. Furthermore, a phylogenetic comparative study revealed low rates of climate niche evolution in plants and animals, on average around 1°C per million years, with the warm and the dry ends of niches evolving even slower than the cold/wet ends [67].

If we want to understand the traits and trade-offs possibly involved in constraining evolution, it may be most efficient to start by identifying the most limiting aspects of climate. For example, Patsiou *et al*. [68] showed that the upper elevational distribution of 100 herbaceous plant species was best explained by growing degree days; the high end of distribution predicted by climate was off by only about 20 m in elevation (deviation between observed elevational limit and that predicted by growing degree days) across the many species. Such cold-end conditions are likely to impose selection on growth and development rates or on cold resistance to lengthen the growing season. Indeed, many studies, especially in ectothermic animals, have found countergradient variation over latitude and elevation, in which genotypes from cold-adapted populations exhibit faster development rate under standardized conditions [69].

At the warm end of the distribution, limiting aspects of climate for herbaceous plants are often associated with snow cover that is too brief (e.g. [70]) or conditions that are too warm or too warm–dry (e.g. [36,68,70]). Patsiou *et al*. [68] showed that the warm end of elevational distribution of 100 plant species was predicted best by summer temperature maximum, except for alpine species for which (too high) temperature minima in early spring was somewhat more important. Again, such meta-level results suggest that important trade-offs are likely to involve shortening-versus-lengthening vital processes that must be completed within a growing season and/or increasing-versus-decreasing the effective duration of the growing season by altering tolerance or resistance to abiotic stress.

Our emphasis on potential trade-offs related to climate adaptation is not meant to imply that biotic interactions are unimportant. For some species, biotic interactions are predicted to drive large-scale distribution, especially if positive interactions such as mutualism and commensalism, or consumer-resource interactions with a positive effect that outweighs the negative effect are important [71]. Furthermore, there has been the tradition of thinking that the warm end of distribution is typically caused by species interactions, while the cold end of distribution is caused by climatic harshness (reviewed in [72]). A recent count study on the importance of abiotic versus biotic factors explaining range limits revealed that the warm end was indeed about equally often explained by abiotic/temperature and biotic factors, while the cold end was often explained by temperature [73]. Furthermore, a study on trees over elevational gradients found that the location of the climatically more stressful end could flip even among different mountain slopes for a given species [74]. Some studies also suggest the importance of interactions between climate and biotic interactions (e.g. [75]). These insights motivate studying the relative importance of both abiotic and biotic factors as well as an interplay between them within study systems.

## 9. Where to next? Studying trade-offs

No study listed in table 1 provided clean evidence for an allocation trade-off by manipulating resources that could be limiting at range edges. Furthermore, hardly any study was set up to detect genetic trade-offs acting within populations (but possibly across environments), a requirement to unequivocally document that a trade-off contributes to range limits. One method that is reliable and informative for measuring microevolutionary genetic trade-offs is to impose selection on one trait and measure correlated responses in other traits ([13]; see [76] for an example). A single generation of selection is sufficient in principle, although several generations may be more informative. The highest response may be revealed not by working with a population from the range edge, but a genetically diverse, central population or by first producing hybrids of divergent lineages (e.g. [21]). The next step is to confirm that selection acts on these traits at range edges *in situ*, or antagonistically across range edges—from within to beyond.

Another approach to measuring genetic trade-offs is a breeding design, and the measuring of relevant traits coupled with fitness measures. In principle, this can be done with any diverse population from the area of distribution, but ideally with populations closer to the range edge. The approach is more likely to detect a trade-off if resources are limited, if the study is performed in the field and if it includes manipulation [13]. Again, an interesting addition is to include sites at the edge and beyond the edge (or corresponding conditions) as this will allow the detection of trade-offs that act across the environmental gradient around range limits. The results can be depicted with fitness maps (fitness on a two-dimensional trait plane) indicating which trait combinations optimize fitness in an environment, or, when separately plotted for within and beyond the range edge, across environments, and the extent and shape of the trade-off. Trade-offs need not be linear, and revealing detailed shape is relevant for assessing evolutionary implications.

However, before diving into such studies, we need to know the relevant traits that may be involved in trade-offs. Section 8 provided some ideas on how to begin. It helps to have a clear idea of the important niche- and range-determining environmental variables, which then suggests a list of fairly integrative candidate traits to study, such as tolerance or resistance to temperature extremes or drought (also see [77]). Existing mechanistic models on functional or physio-chemical constraints potentially relevant to a study organism can suggest further candidates that in the end, when a trade-off is found, may reveal how it acts (e.g. incorporating traits of the leaf and wood economics spectra; see [27]). Candidate traits may then be screened for relevance by studying trait clines across populations (or species) along the gradient. In fact, many trade-offs reported in table 1 have only been studied on this level so far. There is an important caveat to such clinal studies; comparisons should focus *a priori* on differences from the centre to each of the edges, and not from edge to edge. The studies listed in table 1 showed that different trade-offs may be relevant at different range edges. Therefore, reference populations should be those towards the range centres.

In such studies of screening of candidates, care should be taken to details of assessment. Speed of growth and development and resistance traits seem to be regularly involved in trade-offs and may be detected more often if assessed carefully. Aspects of growth are estimated best by repeated size measuring over time and parameter estimation based on a well-supported growth model (e.g. [78]). Furthermore, phenology, growth and development are typically controlled by the environment, and trade-offs involving them may only be revealed under particular temperature conditions (e.g. [21,22]) or under particular day lengths (e.g. [57]). Common garden and laboratory studies need to make sure that environmental conditions simulate those in nature well enough to produce a meaningful response or variability in response depending on provenance.

Many species have range limits with a history of relatively recent range expansion. Range expansions can be associated with genetic drift, accumulation of mutational load and a reduction in vital rates [79,80]. This means that when we compare performance among populations from range centre to range edge, or when we study macroevolutionary patterns of constraint by comparing species sampled at range edges, we need to account for the potential confounding effects of mutation accumulation. This is another reason why conclusive statements on the role of genetic trade-offs are provided best by studies of selection and correlational responses.

In summary, trade-offs may be of general importance in constraining the evolution of niche expansion at range limits. Promising directions for assessing the importance of trade-offs in geographical ranges restricted by climate are determining the type and severity of stress during the harsher time of the year, and estimating the duration of generally benign conditions during the year. Screening for candidate traits is best done by comparing populations within species and using more central populations as a reference. The focal traits are likely to include aspects of resistance/tolerance and speed of growth/development, together with their costs. Testing for environmental trade-offs requires extensive environmental manipulation, and genetic trade-offs ideally involve (artificial) selection to produce the clearest results. We advocate that by assessing the role of trade-offs in the study of range limits rigorously and more quantitatively including at sites in the field, we may be able to answer why range limits establish rather stably. Progress in the field will be of high relevance also to applied sciences including artificial breeding and conservation.

## Data accessibility

This article has no additional data.
